# Bacterial extracellular vesicles modulate epithelial antiviral responses via macrophage-mediated immunomodulation

**DOI:** 10.1186/s12964-026-03003-x

**Published:** 2026-06-24

**Authors:** Jeff Bierwagen, Mosche Lückhof, Miriam Ruth Heindl, Eva Böttcher-Friebertshäuser, Christoph Rummel, Mareike Lehmann, Bernd Schmeck, Anna Lena Jung

**Affiliations:** 1 Institute for Lung Research, Universities of Giessen and Marburg Lung Center, Philipps-University Marburg, German Center for Lung Research (DZL), Marburg, Germany; 2 Institute of Virology, Philipps-University Marburg, Marburg, Germany; 3 Department of Veterinary Physiology and Biochemistry, Justus Liebig University Giessen, Giessen, Germany; 4 Comprehensive Pneumology Center (CPC), Institute of Lung Health and Immunity, Helmholtz Zentrum München, German Center for Lung Research (DZL), Munich, Germany; 5 Institute for Lung Health, Giessen, Germany; 6 Core Facility Flow Cytometry - Bacterial Vesicles, Philipps-University Marburg, Marburg, Germany; 7 Center for Synthetic Microbiology (SYNMIKRO), Philipps-University Marburg, Marburg, Germany; 8 Department of Pulmonary and Critical Care Medicine, Clinic for Respiratory Infections, University Medical Center Marburg, Philipps-University Marburg, Marburg, Germany; 9 Member of the German Center for Infectious Disease Research (DZIF), Marburg, Germany

**Keywords:** Bacterial extracellular vesicles (bEVs), Host-pathogen interaction, Human macrophages, Innate immune response, SARS-CoV-2, Pulmonary infection, Toll-like receptor 4 (TLR4)

## Abstract

Bacterial extracellular vesicles (bEVs) are nano-sized, mostly spherical, double-membrane structures secreted by bacteria throughout their life cycle. In addition to their role in many prokaryotic processes, they can interact with cells of the host immune system, enabling a potential for medical application. This study investigated how bEVs from various pathogenic bacteria modulate human macrophage immune responses and their subsequent ability to protect lung alveolar epithelial cells against SARS-CoV-2 infection.

Primary human blood-derived macrophages were stimulated with bEVs derived from *Legionella pneumophila* (*Lp*), *Klebsiella pneumoniae* (*Kp*), *Escherichia coli* (*Ec*), *Salmonella Typhimurium* (*Sal*) and *Streptococcus pneumoniae* (*Sp*). bEVs from *Kp*, *Ec* and *Sal* strongly induced pro-inflammatory cytokines and interferon-stimulated genes via Toll-like receptor 4-dependent signaling. In contrast, bEVs from *Lp* or *Sp* elicited minimal or no immune response. Conditioned media from *Kp*-, *Ec*- or *Sal*- bEV-stimulated macrophages further influenced Calu-3 epithelial cells, leading to the induction of interferon-stimulated genes. Notably, SARS-CoV-2 propagation tended to be reduced in Calu-3 cells pre-stimulated with conditioned media from *Kp*-bEV- and *Ec*-bEV-treated macrophages, as demonstrated by decreased infectious virus titers in TCID_50_ assays.

These findings provide new insights into macrophage-bEV interactions, demonstrating that gram-negative *Kp*- and *Ec*-derived bEVs induce a potent interferon-dependent antiviral state that can be propagated to lung epithelial cells in vitro. This study highlights a macrophage-driven mechanism of innate immune modulation that extends beyond immune cells to shape antiviral responsiveness in structural cells, providing a conceptual framework for future immunomodulatory or vaccine-oriented strategies.

**Supplementary Information:**

The online version contains supplementary material available at 10.1186/s12964-026-03003-x.

## Background

In the year 2019, the severe acute respiratory syndrome coronavirus type 2 (SARS-CoV-2) newly emerged in the city of Wuhan, China [1]. SARS-CoV-2 was quickly identified as being a member of the *Betacoronavirus* genus within the *Coronaviridae* family and has a rather large, ⁓30 kb long, positive-oriented single-stranded RNA genome [2]. It took the virus only a few months to show its high potential to spread around the world, which culminated in a global pandemic of a magnitude rarely seen before in modern times [3]. According to the World Health Organization, at the time of writing, the global incidence of SARS-CoV-2 has resulted in more than 779 million cumulative infections and more than 7 million deaths worldwide [4]. Even without factoring in such pandemics, pulmonary diseases and infections are one of the leading causes of death worldwide and pose multiple challenges both for patients and health care systems worldwide [5, 6]. After viral lung diseases, the risk of a secondary infection, usually caused by bacterial pathogens, is often increased before the primary illness has fully resolved, which can complicate treatment and raise mortality [7]. Beyond acute co-infections, growing evidence shows that prior microbial encounters can leave lasting effects on host immunity, shaping how tissues respond to later infectious challenges [8]. Importantly, while such clinical scenarios often involve viral infections followed by secondary bacterial complications, prior microbial exposure can shape subsequent immune responses in a sequence-independent manner.

Macrophages are a specialized cell type of the innate immune system and play important roles in many organs. Especially in the lung, which is in constant contact with the external environment, macrophages play a key role in pathogen detection, clearance and the initiation of immune responses [9]. They exhibit remarkable plasticity and, in addition to their other functions, often act as a bridge between the innate and adaptive immune responses [10]. However, macrophages can take on a very different and negative role during some infectious diseases, where they contribute to pathogenicity or are exploited by pathogens directly infecting them, such as *Legionella pneumophila* (*Lp*) [11]. Given that the lungs are in constant exchange with the environment, a high risk of inhaling microorganisms necessitates a tight surveillance of the immune system. At the same time, the lung epithelium must be flexible and thin enough to allow gas exchange, making it vulnerable to unintended damage from immune responses. Taken together, this means that macrophages and lung epithelial cells must maintain a delicate balance between activating and suppressing immune responses to effectively fight pathogens and minimize damage to the lung epithelium to maintain barrier function [12].

Bacterial extracellular vesicles (bEVs) are naturally produced by bacteria during their normal life cycle and are an integral feature of living cells [13]. While the involvement of bEVs in key biological processes, like biofilm formation, nutrient acquisition and interbacterial communication has been demonstrated in some species, other potential roles remain unclear [14]. Structurally, bEVs are nanometer-sized spherical particles (typically 20 to 250 nm) composed of a lipid bilayer carrying a range of bioactive molecules, including proteins, lipopolysaccharides (LPS) or nucleic acids [15]. The ability of bEVs to transport pathogen-associated molecular patterns (PAMPs) and other antigens capable of eliciting host immune responses suggests that bEVs from pathogenic bacteria may influence infection outcomes. While bEVs can have beneficial effects for bacteria, such as promoting their survival, establishing biofilm formation or spreading of virulence factors that increase the susceptibility of cells to infection, they can also enable patrolling immune cells to recognize and respond to an ongoing infection [16]. Gram-negative bacterial bEVs may have particularly strong immunogenic properties due to the presence of LPS on their outer membrane leaflet, mirroring its location in the originating bacteria. These LPS molecules are then recognized by Toll-like receptor 4 (TLR4), a key pattern recognition receptor responsible for detection of PAMPs and the initiation of immune responses [17]. This immune-stimulatory capacity highlights the potential of bEVs as tools for the development of new therapy options or future vaccination approaches [17, 18]. Importantly, not all bEVs carry identical immunostimulatory potential. In particular, structural diversity of LPS among gram-negative bacteria critically influences host recognition and downstream TLR4 signaling [19, 20]. While enterobacterial LPS typically exhibits a hexa-acylated lipid A structure that efficiently activates TLR4-dependent inflammatory and interferon (IFN) pathways, other gram-negative bacteria produce structurally modified LPS variants with reduced or altered TLR4 agonistic activity [21]. Consequently, macrophage responses to bEVs are expected to vary substantially depending on bacterial species, LPS composition, and vesicle-associated pattern recognition receptor ligands. Systematic comparison of bEVs from different bacterial species therefore offers a means to link defined vesicle-associated molecular features to distinct innate immune activation profiles.

Building on our previously published work demonstrating that bEVs can induce macrophage-intrinsic antiviral immune responses [22], the present study was designed to systematically compare bEVs from distinct bacterial species with defined differences in cell envelope composition, innate immune recognition, and macrophage interaction profiles and their subsequent effect on airway epithelial cells. Specifically, we selected gram-negative bacteria producing canonical enterobacterial LPS (*Klebsiella pneumoniae*, *Escherichia coli*, and *Salmonella enterica* serovar Typhimurium), a gram-negative pathogen characterized by structurally atypical LPS and a pronounced tropism for macrophages (*Legionella pneumophila*), and a gram-positive respiratory pathogen lacking LPS altogether (*Streptococcus pneumoniae*).

The bacterial species included in this study were selected to represent diverse classes of bacteria with distinct EV composition and immunostimulatory potential, enabling comparative analysis of sequence-independent bEV-mediated immune signaling rather than modeling specific clinical infection scenarios. The central hypothesis underlying this comparative approach was that the immunostimulatory and antiviral properties of bEVs scale with their capacity to engage TLR4-dependent innate immune signaling pathways in macrophages. We further hypothesized that differential macrophage activation by bEVs would translate into distinct paracrine effects on neighboring lung epithelial cells, thereby modulating their antiviral state and susceptibility to subsequent viral infection. To test this hypothesis, we investigated macrophage responses to bEVs from the selected bacterial species and examined how macrophage-derived soluble mediators influence epithelial IFN signaling and SARS-CoV-2 replication in vitro.

## Materials and methods

### Chemicals and antibodies

RPMI 1640 and DMEM were purchased from Gibco (part of ThermoFisher Scientific, Waltham, USA). GlutaMax™ and fetal calf serum (FCS) were acquired from Life Technologies (Darmstadt, Germany). PBS was provided by anprotec (Bruckberg, Germany). Phorbol 12-myristate 13-acetate (PMA) was bought from Sigma-Aldrich Chemie (Munich, Germany). Lipopolysaccharides (LPS) from *E. coli* serotype O111:B4 was acquired from (Sigma-Aldrich, Burlington, USA), while Normocin, Zeocin, Blasticidin and LPS from *Rhodobacter sphaeroides* (LPS-RS) were from InvivoGen (Toulouse, France). Ruxolitinib was purchased from BIOZOL Diagnostica Vertrieb GmbH (Eching, Germany). All other applied chemicals were of analytical grade and acquired from commercial sources.

### Bacterial culture and bEV purification

Bacterial culture and purification of bEVs was performed as previously described [22, 23]. Briefly, overnight cultures for bacteria were grown on agar plates (buffered charcoal yeast extract agar for *Legionella pneumophila* (*Lp*) Corby, MacConkey for *Klebsiella pneumoniae* (*Kp)*, *Escherichia coli* (*Ec)*, *Salmonella enterica* (*Sal*) serovar Typhimurium and blood agar plates for *Streptococcus pneumoniae* (*Sp*)). For all gram-negative bacteria plated on MacConkey agar (*Kp*, *Ec* and *Sal*), solitary colonies were used to inoculate a 2 ml LB day culture in capped plastic tubes, incubated at 37 °C and 160 rpm until early exponential phase. In the afternoon, 1 ml was transferred into 10 ml fresh sterile LB medium in a 100 ml Erlenmeyer for overnight culture. For *Lp*, bacteria were used for a starter culture in BYE medium in a 15 ml falcon tube (starting OD₆₀₀ of 0.2). *Lp* starter cultures were incubated for approximately 4 h at 37 °C and 160 rpm before use to inoculate the production culture. Start cultures were then used to inoculate liquid media (BYE for *Lp*, LB for *Kp*, *Ec*, *Sal* and THY for *Sp* coming directly from the blood agar plate) and bacteria were incubated to late exponential growth phase. Because growth kinetics differ substantially between bacterial species, incubation times were individually optimized for each strain to ensure harvesting at comparable physiological states. Growth phase was monitored by optical density measurements, and bEVs were consistently isolated at strain-specific time points corresponding to late exponential growth, rather than at fixed incubation times across species (Figure S1). Bacteria and debris were removed by three centrifugation steps (4,800xg, 20 min, 4 °C) and subsequent sterile filtration through a 0.22 μm pore size filter. The filtered supernatant was concentrated to 500 µl using 100 kDa molecular weight cut-off filters (Merck KGaA, Darmstadt, Germany). Concentrated supernatants were then further processed by size exclusion chromatography (SEC) by loading onto qEVoriginal/ 70 nm Gen 2 columns (IZON Science LTD, Lyon, France). Following the manufacturer´s protocol, IZON columns were equilibrated with 10 ml sterile and 0.1 μm pore size filtered PBS (Cytiva Hyclone, South Logan, USA) prior to loading with vesicle concentrates. When fractions of 500 µl were collected, vesicles eluted into fractions 7–12, which were pooled and concentrated to 500 µl, using 100 kDa molecular weight cut-off filters (Merck KGaA, Germany). Particle concentration of bEV preparations was determined by nano-flow cytometry measurements (nFCM; NanoFCM Co., Ltd, Nottingham, UK) and aliquots were stored at -80 °C until used in experiments. Additionally, all bEV preparations were routinely checked for bacterial contamination, by plating an aliquot on blood agar plates and incubating them at 37 °C for several days. Preparations were only used for experiments if no colony growth was detected.

### Isolation, stimulation and culture of blood-derived macrophages

Human blood-derived macrophages (BDMs) were isolated from buffy coats, obtained from the Department of Transfusion Medicine at the University Hospital of Giessen and Marburg. Buffy coats were collected from healthy adult donors (≥ 18 years), and no specific inclusion criteria regarding sex or age were applied. All donors gave informed written consent (Ethics approval number: 161/17). First, peripheral blood mononuclear cells were isolated by diluting buffy coats with PBS (ratio 1:1) and centrifuging in a density gradient medium with Histopaque-1077 (Sigma-Aldrich, Burlington, USA). After multiple washes of the isolated cells with PBS, monocytes were enriched and purified by positive selection, using the MACS system and CD14 microbeads (both Miltenyi Biotec, Germany). Enriched monocytes were then differentiated into blood-derived macrophages by culturing in RPMI 1640 medium supplemented with 10% heat-inactivated FCS and 15 ng/ml hGM-CSF (PeproTech, Rocky Hill, USA) for 7 days. An additional supplementation of hGM-CSF was performed on day 4 to support macrophage differentiation. After differentiation, cells were stimulated by switching to a fresh growth medium containing bEVs at a calculated concentration of 100 bEVs per macrophage (MOV; multiplicity of vesicles) or, in the case of controls, fresh media without bEVs. BDMs were stimulated for 20 h, after which the supernatant was collected and RNA from the attached cells was isolated for further experiments. The 20 h stimulation period for macrophages was optimized as a standardized experimental parameter to allow robust cytokine and interferon induction while maintaining cell viability.

### ELISA

Supernatants of stimulated BDMs were used to quantify the cytokines IL-8/CXCL8, IL-1β and IFN-β using the commercially available DuoSet ELISA kits from R&D Systems (Wiesbaden, Germany) according to the manufacturer´s protocols. The lower limits of detection were 31.2 pg/mL for CXCL8, 3.91 pg/mL for IL-1β, and 7.81 pg/mL for IFN-β.

### RNA isolation and reverse transcription

RNA from non-infection experiments was isolated from cells using the phenol-chloroform extraction method as previously described [23]. For all infection experiments, RNA isolation was performed using the QIAGEN Mini RNeasy Kit (QIAGEN GmbH, Germany) according to the manufacturer´s protocol for animal cells.

Total RNA was reverse transcribed into cDNA using a high-capacity cDNA reverse transcription kit (ThermoFisher Scientific, Waltham, USA) with random hexamer primers, following the manufacturer´s instructions.

### Quantitative real time PCR

Quantitative Real-Time PCR was performed on a QuantStudio™ 3 instrument (ThermoFisher Scientific, Waltham, USA) using Luna^®^ Universal qPCR Master Mix (New England Biolabs, Ipswich, USA) and gene-specific primer pairs. Relative expression levels were calculated using the 2^−ΔΔCt^ method, normalized to a housekeeping gene and to the respective control cells [24]. The primer pairs used were:

OASL: fwd: 5‘-CAGCAATCAACCCCAAACCA-3‘, rev: 5‘-TGGTATGGGGAAGCTGGAAG-3‘.

ISG20: fwd: 5‘-GATCACCGATTACAGAACCCG-3‘, rev: 5‘-TTCAGTGCCTGGAAGTCGTG-3‘.

Mx1: fwd: 5‘-GGGCTTTGGAATTCTGTGGC-3‘, rev: 5‘-CCTTGGAATGGTGGCTGGAT-3‘.

IFIT1: fwd: 5‘-ATGCAGGAAGAACATGACAACC-3‘, rev: 5‘-TCTGGACACTCCATTCTATAGCG-3‘.

RPS18: fwd: 5’-GCGGCGGAAAATAGCCTTTG-3‘, rev: 5‘-GATCACACGTTCCACCTCATC-3‘.

SARS-CoV-2 E-gene: fwd: 5‘-ACAGGTACGTTAATAGTTAATAGCGT-3‘, rev: 5‘-ATATTGCAGCAGTACGCACACA-3‘.

### nFCM

A Nano Analyzer (NanoFCM Co., Ltd, Nottingham, UK) equipped with a 488 nm laser was used for concentration and size measurements of bEV preparations. Prior to measurements, the instrument was calibrated with 250 nm polystyrene beads (NanoFCM Co.) at a defined concentration of 2.16 × 10^10^ particles/ml, which were used as a reference for particle concentration measurements. Additionally, monodisperse silica beads (NanoFCM Co.) specifically designed to contain four different particle sizes were used as a size reference standard for bEV particle size measurements. Background signals were determined by measuring freshly filtered TE buffer (0.2 μm pore size filter; pH = 8) and subtracted from the measurements. Data for size distribution histograms were collected by continuous 1 min measurements with a sample pressure of 1 kPa. bEV samples were diluted with freshly filtered TE buffer (0.2 μm) to obtain particle counts between 2,000 and 12,000 events, which is the optimal range for the instrument. Final particle concentrations and size distributions were calculated using the NF Profession software (V2.3) supplied with the nFCM instrument.

### Endotoxin quantification

Endotoxin levels in bEV preparations were quantified using the Pierce™ Chromogenic Endotoxin Quant Kit (ThermoFisher Scientific) according to the manufacturer’s instructions. Briefly, bEV samples were serially diluted in provided endotoxin-free water and assayed in parallel with a standard curve generated using purified *E.coli* O111:B4 LPS (Sigma-Aldrich). Absorbance was measured as specified by the kit protocol, and endotoxin concentrations were calculated based on the LPS standard curve. Endotoxin levels detected in bEV preparations were normalized to the number of vesicles analyzed and are reported relative to the LPS reference.

### Western blot

Bacterial extracellular vesicle (bEV) preparations were analyzed by Western blotting as previously described [22]. Equal numbers of vesicles (1 × 10⁸ bEVs per lane) were resuspended in Laemmli sample buffer and denatured (95 °C for 5 min) prior to separation by SDS-PAGE. Proteins were transferred to nitrocellulose membranes and probed with an antibody directed against the outer membrane protein A (OmpA; abbexa #abx110631). Immunoreactive bands were visualized using ECL.

To confirm equal sample loading, Coomassie staining of the corresponding SDS-PAGE gel was performed using Roti^®^-Blue (Carl Roth GmbH + Co. KG, Karlsruhe, Germany).

### Cell culture for THP-1 and THP-1 dual™ and calu-3 cell lines

The human cell lines THP-1 and THP-1 Dual™ cells were obtained from the American Type Culture Collection and InvivoGen (Toulouse, France), respectively. Cells were maintained in RPMI 1640 (supplemented with 10% heat-inactivated FCS, 1% GlutaMax™, 1 mM sodium pyruvate and 100 µg/ml Normocin™) and incubated at 37 °C incubators (Heracell™ 240i, ThermoFisher Scientific) in a 5% CO_2_ atmosphere. Cell numbers were kept between 2 × 10^5^ and 1 × 10^6^ cells/ml. Additionally, THP-1 Dual™ cells were kept under selection pressure by the addition of 10 µg/ml Blasticidin and 100 µg/ml Zeocin antibiotics (both InvivoGen) at every second passage. Cells were used for experiments only until they reached a number of 20 subculturing procedures. Calu-3 cells were grown in DMEM (supplemented with 10% heat-inactivated FCS and 1 mM sodium pyruvate) in a 37 °C incubator with a 5% CO_2_ atmosphere. Calu-3 cells were subcultured in fresh culture flasks when they reached approximately 90% confluence. For LPS-RS experiments, Standard *Rhodobacter sphaeroides* LPS, which antagonizes TLR4 signaling but may also engage TLR2 due to the presence of additional bacterial components, was used for inhibition experiments. THP-1 Dual™ cells were differentiated into macrophage-like cells with 20 nM PMA overnight. Cell stimulation with bEVs was performed for 20 h at a calculated level of 1,000 MOV as previously described [22]. LPS-RS was incubated in the corresponding wells for 1 h prior to bEV stimulation.

### QUANTI-Luc™ assay

Detection of secreted Lucia luciferase activity (interferon reporter) from stimulated THP-1 Dual™ cells was assessed via QUANTI-Luc™ assay (InvivoGen) according to the manufacturer´s protocol. Briefly, cell culture supernatants were added to white flat-bottomed 96-well plates (BRAND GmbH & Co. KG, Wertheim, Germany). QUANTI-Luc™ assay reagent was thawed and added to each well. Luminescence measurement was started immediately with a 0.1 s read time in a microplate reader (Infinite F200 Pro, Tecan Life Sciences, Männedorf, Switzerland).

### Proximity ligation assay

To investigate protein-protein interactions between cells and bEVs, the Duolink^®^ proximity ligation assay (Merck KGaA) was performed according to the manufacturer´s protocol. Cells were seeded on coverslips and differentiated with 20 nM PMA one day before the experiment. The next day, cells were stimulated with bEV_*Ec*_ for 1 h or left untreated as a control, before the medium was replaced with paraformaldehyde solution (4%, pH = 7.2) for 15 min at room temperature (RT) to fix the cells. The fixed cells were then permeabilized with Triton X-100 (0.1% in PBS) for 10 min at RT. The provided Duolink^®^ blocking solution was mixed with 2.5 µg of the human BD Fc Block™ reagent (BD Biosciences, Franklin Lakes, USA) and cells were blocked by incubation at 37 °C for 1 h. After blocking, cells were incubated with primary antibodies targeted against human TLR4 (rabbit antibody, diluted 1:200 in antibody diluent, from Sigma-Aldrich, Burlington, USA) and a second primary antibody targeted against *E. coli* LPS (mouse antibody, diluted 1:200 in antibody diluent, from Abcam, Cambridge, UK) for 1 h at RT. Cells were washed twice with wash buffer (provided in the Duolink™ kit). The PLUS and MINUS probes were vortexed and mixed with antibody diluent in a ratio of 1:5 to create the probe mix, which was added to the cells and incubated at 37 °C for 1 h. After further washing, probe ligation was initiated by adding the ligase mix to the samples and incubating at 37 °C for 30 min. The amplification step was then performed by adding amplification solution to the samples and incubating at 37 °C for 100 min in the dark. After two final washes with the supplied wash buffer B, coverslips were mounted using the DAPI-containing mounting medium also provided with the Duolink™ kit. Analysis was performed on a Leica Stellaris 8 confocal microscope (Leica Microsystems, Wetzlar, Germany) using LAS-X (version 4.7.0.28176) and ImageJ (version 1.54f) software.

### Infection of Calu-3 cells with SARS-CoV-2

Calu-3 cells were seeded in 24-well plates and allowed to adhere overnight. The next day, they were stimulated with cell culture supernatants obtained from bEV-stimulated BDMs. Prior to stimulation of Calu-3 cells, BDM supernatants were mixed with fresh growth medium (DMEM + 10% heat-inactivated FCS) in a 1 + 1 ratio to ensure that the cells were also provided with fresh nutrients. After 20 h of stimulation, a time point chosen to allow establishment of an IFN-dependent antiviral state in epithelial cells prior to viral infection, the medium was changed to infection medium (DMEM + 2% heat-inactivated FCS) and Calu-3 cells were infected with severe acute respiratory syndrome coronavirus type 2 (SARS-CoV-2) at a multiplicity of infection (MOI) of 0.01 for 24 h. The inoculum was removed 1 h after the start of the experiment and replaced by fresh medium. Supernatants were collected for TCID_50_ experiments and RNA was harvested for gene expression analyses by quantitative Real-Time PCR. All infection experiments with SARS-CoV-2 were performed in the BSL-3 laboratory facility at Philipps-University Marburg.

### TCID_50_ assay

The TCID_50_ assay was used to quantify the titer of infectious particles in SARS-CoV-2 preparations and infection experiments. Calu-3 cells were seeded in 96-well plates and incubated until they reached confluency. Supernatant from the infection experiments was diluted in a 5-fold dilution series on top of the Calu-3 cell layer and the plates were incubated for 4 days. Infected wells were identified by microscopic detection of the appearance of a cytopathic effect in the cell monolayer, and the Spearman-Kärber algorithm was used to calculate the dose of infectious particles/ml [25]. At least four technical replicates were performed per sample.

### Statistics

Data are presented as mean ± SEM for at least three biologically independent experiments. Statistical analyses and graph generation were performed using Prism 10.4.1 (GraphPad, La Jolla, USA). Prior to applying parametric tests, data distributions were assessed for normality using the Shapiro-Wilk test. For comparisons involving more than two groups, one-way ANOVA was performed, followed by Tukey’s multiple comparisons post-hoc test. P-values ≤ 0.05 were considered statistically significant. Tests were performed against the corresponding controls unless otherwise indicated.

## Results

### Macrophages release pro-inflammatory cytokines and induce ISGs upon stimulation with bEVs

In this study, isolated bEVs from different bacterial species were investigated for their immunomodulatory properties. The bacteria *Legionella pneumophila* (*Lp*), *Klebsiella pneumoniae* (*Kp*), *Escherichia coli* (*Ec*), *Salmonella Typhimurium* (*Sal*) and *Streptococcus pneumoniae* (*Sp*) were used to isolate the respective bEVs. Size distribution, mean size and particle concentration were determined by nFCM, showing a mean bEV size of 66–75 nm and comparable mean concentrations for all bacterial species studied (Fig. S2 A-B). To further validate the bEV preparations, endotoxin content and the presence of gram-negative outer membrane components were assessed. Endotoxin levels were detected within a comparable range across bEV preparations derived from the gram-negative bacteria (*L. pneumophila*, *K. pneumoniae*, *E. coli*, and *S. Typhimurium*), whereas endotoxin levels in bEV_*Sp*_ were negligible (Fig. S3A). Western blot analysis confirmed the presence of outer membrane protein A (OmpA) in *Enterobacteriaceae* bEV preparations but not in bEV_*Lp*_ and bEV_*Sp*_ (Fig. S3B). Together with the particle size and concentration measurements, these analyses validate the integrity and expected key molecular features of the bEV preparations used for subsequent macrophage stimulation experiments.

Human BDMs were isolated and differentiated from buffy coats and used as a primary cell model for experiments. To define an appropriate stimulation dose, dose–response experiments were performed in primary human BDMs using increasing multiplicities of vesicles (MOV 10–10,000). Pro-inflammatory activation was assessed by measuring CXCL8 and Mx1 expression, while cytotoxicity was evaluated by LDH release. These experiments demonstrated that low-dose stimulation (MOV 10) did not induce measurable activation, whereas higher doses (MOV ≥ 1,000) resulted in increased cytotoxicity. Importantly, MOV 100 consistently induced robust pro-inflammatory responses without compromising cell viability and did not substantially differ from higher doses in terms of activation (Fig. S4A-C). Based on these results, MOV 100 was selected as the working concentration for subsequent experiments.

**Fig. 1:**
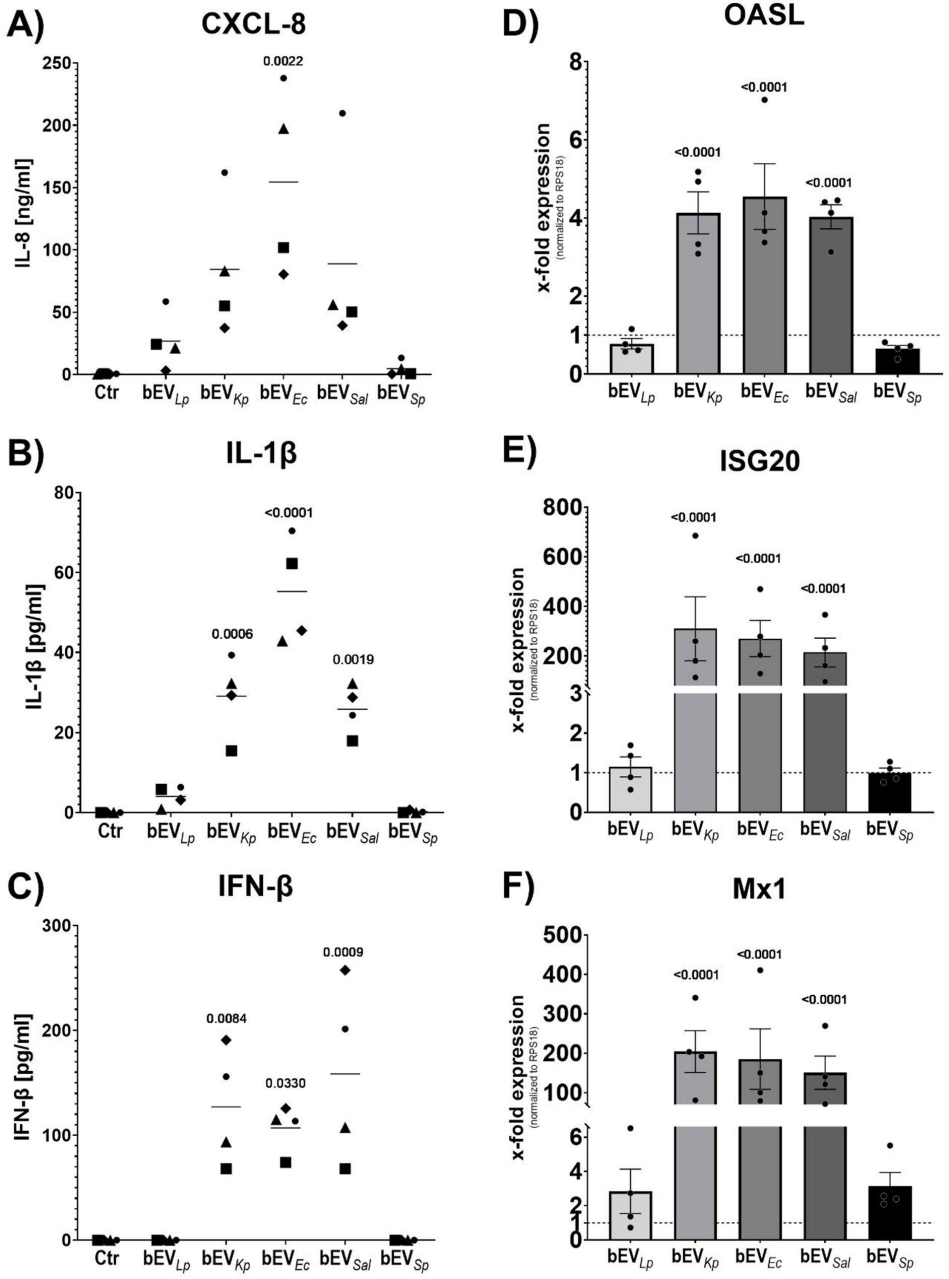
bEVs from gram-negative bacteria induce a pro-inflammatory response in primary human macrophages. **A**-**F** Differentiated blood-derived macrophages (BDMs) were stimulated with bacterial extracellular vesicles (bEVs from *Legionella pneumophila* – *Lp*; *Klebsiella pneumoniae* – *Kp*; *Escherichia coli* – *Ec*; *Salmonella enterica* serovar Typhimurium – *Sal* and *Streptococcus pneumonia* - *Sp*) for 20 h or left untreated as control (Ctr). **A**-**C** The release of the cytokines CXCL8 (**A**), IL-1β (**B**) and IFN-β (**C**) into the cell culture supernatant was measured using ELISA. The lines represent the mean values, while the symbols represent values obtained from each individual donor. **D**-**F** Expression of the interferon-stimulated genes OASL (**D**), ISG20 (**E**), and Mx1 (**F**) was determined by qPCR. Results were normalized to RPS18 and are shown relative to untreated control cells (Ctr), which are indicated as a dashed line. Bars represent mean values + SEM. Statistics: One-way ANOVA followed by Tukey’s multiple comparisons test **A**-**F**. Statistical comparisons were performed against the untreated control condition (Ctr). Exact p-values are indicated in the graphs. Data are shown as mean ± SEM; each symbol represents one biological replicate; *n* = 4

Stimulation of BDMs with bEV_*EC*_ for 20 h, resulted in a significantly increased release of the cytokine CXCL8 compared to unstimulated control cells (Fig. 1A). Furthermore, stimulation of cells with bEVs from *Lp*, *Kp*, and *Sal*, also showed a tendency to induce CXCL8 secretion, although this was not statistically significant. Stimulation of BDMs with bEV_*Sp*_ resulted in CXCL8 levels below the lower limit of detection (Fig. 1A). Investigation of the release of the cytokine IL-1β showed a significant secretion of this cytokine when cells were stimulated with bEVs from *Kp*, *Ec* and *Sal* (Fig. 1B). While a minimal amount of IL-1β was measured after stimulation of cells with bEV_*Lp*_, no secretion was detected when cells were stimulated with bEV_*Sp*_ (Fig. 1B). Essentially the same result was observed when detecting the release of the interferon IFN-β, with the notable exception that stimulation of BDMs with bEV_*Lp*_ resulted in IFN-β levels below the lower limit of detection (Fig. 1C).

As the release of interferons, such as IFN-β, is generally followed by the induction of numerous downstream genes, known as interferon-stimulated genes (ISGs), the induction of a selection of these ISGs was further investigated using qPCR. The expression of *OASL*, *ISG20* and *Mx1* in BDMs was significantly induced by stimulation with bEVs of *Kp*, *Ec*, and *Sal* (Fig. 1D-F). Conversely, the stimulation of BDMs with bEVs from *Lp* or *Sp* did not result in altered expression of these ISGs compared to unstimulated control cells (Fig. 1D-F). These experiments indicate a pro-inflammatory activation of macrophages when the cells recognize bEVs from *Kp*, *Ec* and *Sal*, leading to the release of cytokines and interferons and subsequent induction of ISGs. In contrast, the activation pattern and the release of cytokines and interferons were markedly restricted or absent when macrophages encounter bEVs from *Lp* or the gram-positive bacterium *Sp*.

### TLR4-dependent sensing of vesicle-associated LPS mediates interferon signaling in macrophages

**Fig. 2:**
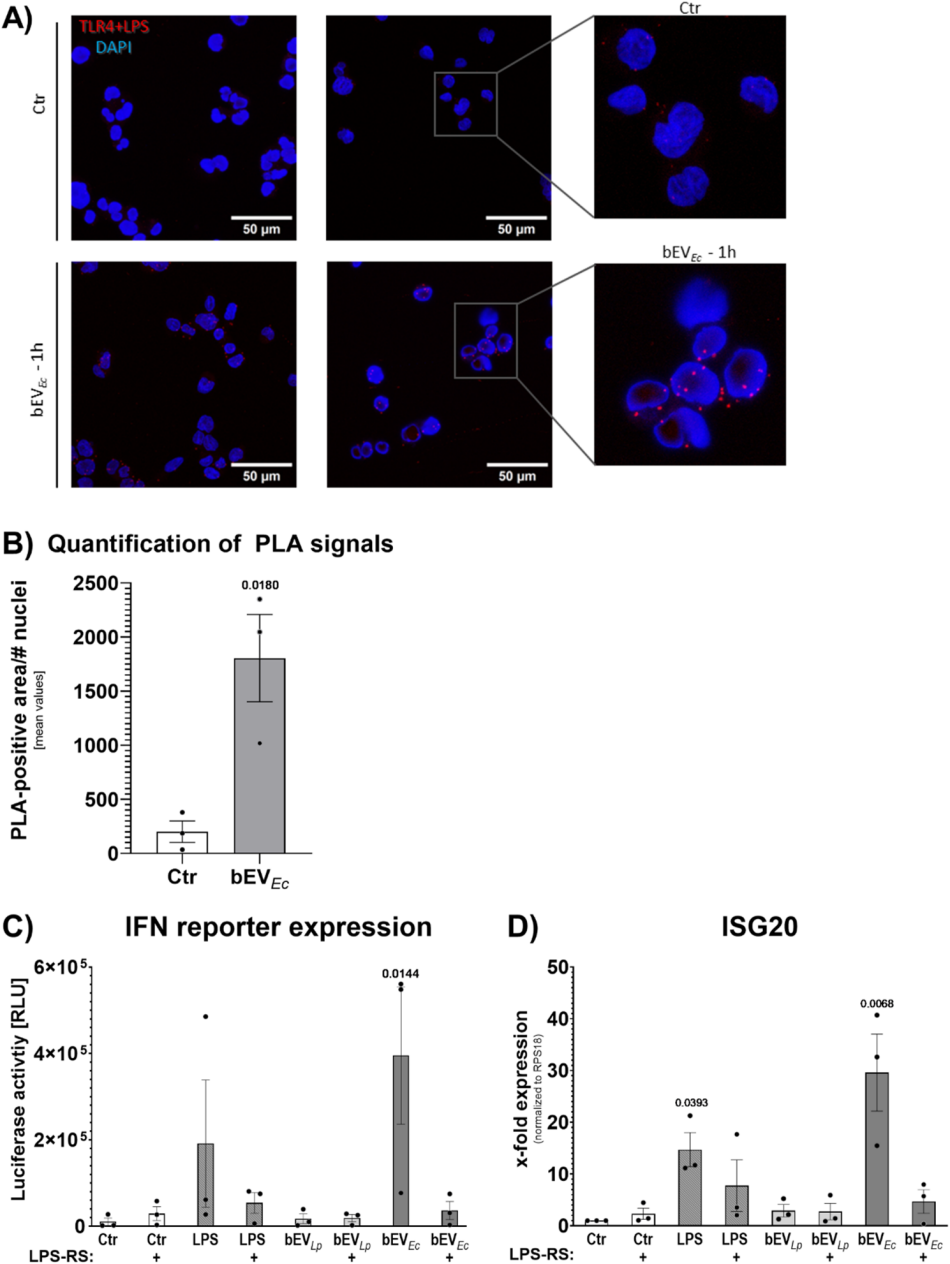
Co-localization shows direct interaction between bEV-located LPS and TLR4 and can be blocked by inhibiting TLR4. **A** Differentiated THP-1 cells were left untreated as control (Ctr) or stimulated with bacterial extracellular vesicles from *Escherichia coli* (bEV_*Ec*_) for 1 h, fixed, and examined by proximity ligation assay (PLA) using TLR4 and LPS antibodies. Fluorescence images were acquired as z-stacks using a confocal laser scanning microscope. Red fluorescent dots show colocalization between TLR4 and LPS, while blue areas show DAPI-stained nuclei. Scale bar = 50 μm. Depicted are representative z-projections derived from corresponding z-stacks. **B** Quantification of PLA-positive fluorescence area divided by the number of nuclei per z-projection image from (**A**). Bars represent the mean values of three pictures per condition. (C + D) THP-1 Dual™ cells were stimulated with lipopolysaccharides (LPS) or bacterial extracellular vesicles from *Legionella pneumophila* (bEV_*Lp*_) or bEV_*Ec*_ for 20 h. Additionally, cells were pre-treated for 1 h either with (+) or without LPS-RS (LPS from *Rhodobacter sphaeroides*), which acts antagonistically on TLR4. **C** Luciferase assay measuring the secreted IFN reporter from THP-1 Dual™ cells. Values are shown in relative luminescence units (RLU) obtained by using the QUANTI-Luc™ detection reagent. **D** ISG20 expression was determined by qPCR. Bars represent the mean values of three independent experiments + SEM. Statistics: Unpaired t-test comparing untreated control cells (Ctr) and bEV_*Ec*_-treated cells (**B**), or One-way ANOVA followed by Tukey’s multiple comparisons test with comparisons performed against the untreated control condition (Ctr) (C + D). Exact p-values are indicated in the graphs; *n* = 3. Symbols represent replicates **B**-**D**

To mechanistically support the hypothesis that vesicle-associated LPS is sensed via TLR4 on macrophages, we first examined the spatial proximity between bEV-derived LPS and macrophage TLR4 using proximity ligation assays (PLA). Differentiated THP-1 cells were stimulated with *Escherichia coli*-derived bEVs (bEV_*Ec*_) for 1 h and subsequently analyzed by PLA using antibodies against LPS and human TLR4. This approach allows detection of molecular interactions occurring within less than 40 nm. Stimulation with bEV_*Ec*_ resulted in the appearance of numerous discrete PLA-positive fluorescent signals, indicating close spatial association between vesicle-associated LPS and macrophage TLR4, whereas unstimulated control cells showed only minimal background signal (Fig. 2A). Quantification of the PLA-positive area normalized to the number of nuclei per image confirmed a significant increase in signal upon bEV_*Ec*_ stimulation compared to controls (Fig. 2B). This close spatial association between vesicle-associated LPS and TLR4 provides a mechanistic basis for subsequent functional analyses of downstream interferon signaling.

Consistent with this physical interaction, we next investigated whether TLR4 engagement is functionally required for bEV-induced interferon responses in macrophages. To this end, macrophage-like THP-1 Dual™ reporter cells were stimulated with recombinant LPS, bEV_*Lp*_ or bEV_*Ec*_, either in the presence or absence of the TLR4 antagonist LPS from *Rhodobacter sphaeroides* (LPS-RS). Stimulation with recombinant LPS or bEV_*Ec*_ induced robust activation of the interferon reporter, whereas bEV_*Lp*_ did not elicit a detectable response (Fig. 2C). Pre-treatment with LPS-RS abrogated interferon reporter activation in response to both recombinant LPS and bEV_*Ec*_, demonstrating TLR4 dependence of the observed signaling.

These findings were further corroborated at the transcriptional level, as bEV_*Ec*_-induced expression of the interferon-stimulated gene ISG20 was reduced upon pharmacological interference with LPS-dependent TLR4 signaling, while bEV_*Lp*_ stimulation had no effect on ISG20 expression (Fig. 2D). Consistent with this, partial inhibition of the response was also observed upon LPS neutralization using polymyxin B (Fig. S5), further supporting a role for LPS-containing components. Together, these data demonstrate that vesicle-associated LPS from enterobacterial bEVs engages TLR4 on macrophages and functionally drives downstream interferon signaling, whereas bEVs derived from *L. pneumophila* fail to activate this pathway.

### ISGs are induced in lung epithelial cells by macrophage derived cytokines as reaction to bEVs

**Fig. 3:**
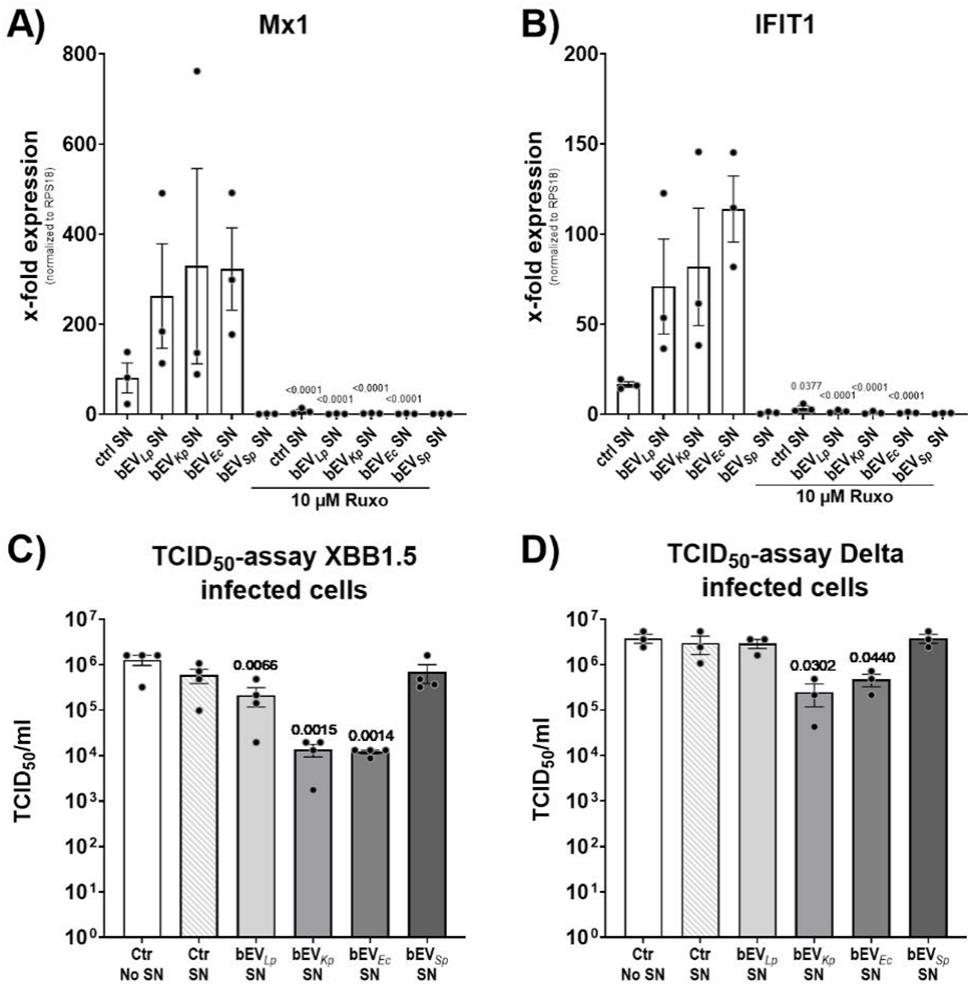
Supernatants from bEV-stimulated BDMs induce ISGs in Calu-3 cells and confer antiviral properties in subsequent infection with SARS-CoV-2. **A**-**D** Calu-3 cells were stimulated with supernatants obtained from BDMs stimulated with bEVs from *Legionella pneumophila* (bEV_*LP*_ SN), *Klebsiella pneumoniae* (bEV_*Kp*_ SN), *Escherichia coli* (bEV_*Ec*_ SN) or *Streptococcus pneumoniae* (bEV_*Sp*_ SN) or from unstimulated control BDMs (Ctr SN), as indicated on the x-axis. Both stimulation steps were performed for 20 h each. Where indicated, Calu-3 cells were additionally treated with 10 µM Ruxolitinib (Ruxo). (A + B) Relative expression of Mx1 (**A**) and IFIT1 (**B**) was determined by qPCR and normalized to RPS18. Data are shown as x-fold change relative to untreated Calu-3 cells from three independent experiments (not shown in the graph). Bars represent mean ± SEM. Symbols indicate biological replicates derived from independent BDM donors. (C + D) Calu-3 cells were stimulated as described above, followed by infection with SARS-CoV-2 Omicron XBB1.5 (**C**) or Delta (**D**) variants at a multiplicity of infection (MOI) of 0.01. After 24 h, supernatants were collected and analyzed for viral titers using the TCID_50_ assay. Infectious particle concentrations in particles/ml were determined using the Spearman-Kärber algorithm. Bars represent mean ± SEM from 3–4 independent experiments. Symbols indicate biological replicates. Statistics: One-way ANOVA followed by Tukey’s multiple comparisons test. For panels **A** and **B**, statistical comparisons were performed between matched conditioned supernatant treatments in the absence or presence of 10 µM Ruxolitinib (Ruxo). For panels **C** and **D**, statistical comparisons were performed against the conditioned supernatant control (Ctr SN). Exact p-values are indicated in the graphs; *n* = 3–4

Cytokines are well-established activators of cellular immune responses, acting in both an auto- and paracrine manner. To investigate whether macrophages activated by bEV recognition also stimulate cellular immune responses in surrounding cells, we collected cell culture supernatants from bEV-stimulated BDMs. In a second step, the conditioned supernatants were used to stimulate Calu-3 cells, a well-established model for studying lung epithelial cells in vitro, particularly in the context of viral lung infection. Calu-3 cells were treated with conditioned supernatants from BDMs for 20 h and the induction of a panel of four ISGs (*OASL*, *ISG20*, *Mx1* and *IFIT1*) was assessed. Supernatants conditioned from bEV_*Kp*_- and bEV_*Ec*_-stimulated macrophages resulted in the strongest induction of all four ISGs, whereas conditioned supernatants from unstimulated or bEV_*Sp*_-stimulated macrophages showed lower or no detectable induction of ISG expression in Calu-3 cells as determined by qPCR (Fig. 3A + B, Fig. S6 A + B). The observed cellular responses to conditioned supernatants from bEV_*Kp*_- and bEV_*Ec*_-stimulated macrophages showed a trend of reduction, in case of *OASL* and *ISG20* (Fig. 3A) or significantly blocked for *Mx1* and *IFIT1* (Fig. 3B) when Calu-3 cells were pre-treated with the Janus kinase inhibitor Ruxolitinib, which blocks the pathway activated by the interferon-α/β receptor (IFNAR). These experiments indicate that macrophage-derived interferons induced by bEVs from gram-negative bacteria mediate an interferon-dependent paracrine antiviral response state in Calu-3 lung epithelial cells.

### Lung epithelial cells show reduced replication potential of SARS-CoV-2 after activation by bEV-sensing macrophages

Having established that bEV-recognizing macrophages induce an immune response, we wondered if this would be sufficient to affect cells in the context of viral lung infection. The Calu-3 cell line is particularly well suited for in vitro infection studies with SARS-CoV-2 as it expresses the entry receptor ACE2 as well as the important protease TMPRSS2 and is widely used for this purpose [26]. Calu-3 cells were treated with conditioned media from bEV-stimulated BDMs and subsequently infected with either a SARS-CoV-2 Omicron XBB1.5 or Delta lineage virus at an MOI of 0.01 for 24 h. Supernatants and cellular RNA were collected from infected cultures; infectious progeny virus titers were determined using TCID_50_ assays, and viral RNA levels were quantified by qPCR targeting the SARS-CoV-2 E gene.

Infection of stimulated Calu-3 cells with the Omicron XBB1.5 lineage virus resulted in a 2-log reduction in viral titers for bEV_*Kp*_ or bEV_*Ec*_ conditioned supernatants. According to the qPCR data, intracellular viral RNA was reduced to approximately 20–30% of the basal level in infected and unstimulated control cells, indicating an inhibitory effect on viral replication in these stimulated cells (Fig. S7 A). BDM supernatants from bEV_*Lp*_ conditioned supernatants reduced viral titers by about 1-log, while the other conditions investigated (unstimulated control and bEV_*Sp*_) did not reduce viral titers significantly compared to Calu-3 cells that were not treated with bEVs (Fig. 3C). However, the RNA data for these cells demonstrated greater variability between experiments, yet the mean values remained closer to the baseline level determined in unstimulated, infected control cells (Fig. S7 A). When stimulated Calu-3 cells were subsequently infected with a Delta lineage SARS-CoV-2 virus, viral titers were reduced by 1-log for the bEV_*Kp*_- and bEV_*Ec*_- conditioned supernatants (Fig. 3D). Furthermore, viral RNA levels, as determined by qPCR targeting the viral E gene, showed a reduction of intracellular viral RNA to approximately 20–30% of the base level determined in unstimulated, infected control cells (Fig. S7 B). All other conditions (unstimulated control, bEV_*Lp*_ and bEV_*Sp*_) showed similar viral titers and amounts of viral RNA (Fig. 3D and Fig. S7 B). Taken together, these experiments support the concept that macrophage-derived interferons induced by bEVs from gram-negative bacteria, particularly *Kp* and *Ec*, promote an interferon-dependent antiviral state in lung epithelial cells. This response was associated with reduced SARS-CoV-2 replication and lower release of infectious viral particles in vitro.

## Discussion

In this study, we report that bEVs from the gram-negative bacteria *Klebsiella pneumoniae* (*Kp*), *Escherichia coli* (*Ec*) and *Salmonella enterica* serovar Typhimurium (*Sal*) are strong activators of a TLR4-dependend immune response in primary human macrophages in vitro. In contrast, bEVs from *Legionella pneumophila* (*Lp*) and the gram-positive bacterium *Streptococcus pneumoniae* (*Sp*) elicited markedly weaker or no detectable macrophage activation under the conditions tested. This immune response was able to propagate a state of “pre-activation” to lung epithelial cells via cytokines and interferons, which in turn was associated with reduced viral replication in epithelial cells. The present study addresses macrophage-driven paracrine immune communication and demonstrates that bEV-activated macrophages can impose an antiviral state on lung epithelial cells through paracrine interferon-dependent signaling. While the immunogenic nature of gram-negative bEVs and their use as vaccine platforms are well established [27, 28], less is known about how bEVs from different bacterial species differentially shape macrophage-mediated paracrine antiviral signaling.

Primary human BDMs responded to stimulation with *Kp*-, *Ec*-, and *Sal*-derived bEVs with secretion of the cytokines CXCL8, IL-1β and IFN-β and subsequent induction of the interferon-stimulated genes OASL, ISG20, and Mx1, demonstrating activation of pro-inflammatory and interferon-associated signaling pathways. This effect is consistent with several reports in the literature [17, 29–32]. In contrast to studies observing interactions between host cells and bEVs from a single pathogenic bacterium, we established an experimental setting comparing the immunostimulatory capacities of bEVs derived from multiple pathogenic bacteria. Secretion of cytokines as well as induction of the ISGs studied were reduced or below the lower limit of detection when macrophages were stimulated with bEVs derived from *Lp* or *Sp*. Importantly, the limited response observed for *S. pneumoniae*-derived bEVs in this study does not imply an absence of immunomodulatory capacity, but rather reflects engagement of alternative pattern recognition receptors distinct from TLR4-dependent signaling pathways. One feature distinguishing these two bacteria from the others in our study is their lack of efficient TLR4 recognition. Specifically, while *Sp* as a gram-positive bacterium lacks LPS altogether, *Lp*-LPS has an altered molecular structure, representing a well-characterized example of atypical LPS with reduced TLR4 agonistic activity and thereby interfering with its TLR4 recognition [33, 34]. Nonetheless, TLR4 remains a key receptor for the recognition of gram-negative bacteria, usually through their LPS molecules, and activates both MyD88- and TRIF-dependent pathways. This dual-pathway activation is a unique feature of TLR4, culminating in pro-inflammatory and IFN-mediated antiviral signaling [35]. TLR2, a receptor primarily associated with recognition of gram-positive cell wall components, has also been implicated in sensing the structurally altered *Lp*-LPS [36, 37]. This claim, however, was recently challenged by Grigoryeva et al., who showed that the combined involvement of TLR2-, TLR3-, TLR4- and TLR5-signaling was required for a full human macrophage cytokine response to whole *Legionella pneumophila* bacteria [38]. In contrast, our study focuses specifically on vesicles released by the bacteria. Activation of TLR3-dependent IFN responses requires delivery of RNA ligands to endosomal compartments, highlighting that engagement of RNA-sensing PRRs depends not only on ligand presence but also on vesicle-associated trafficking and processing mechanisms. Consistent with this notion, we did not observe induction of IFN-stimulated genes in macrophages exposed to bEV_*Lp*_, suggesting limited engagement of IFN-inducing PRRs by these vesicles despite the known multi-receptor sensing of whole *Legionella* bacteria. Notably, *Lp* is the only bacterium in our panel that is specialized to infect macrophages and may therefore have evolved vesicle-associated mechanisms to modulate the macrophage immune responses, which would be distinguishing it from the other gram-negative bacteria in our setting [39].

PLA has previously been used to show colocalization of bEV-mediated LPS and caspase-11, a well-known intracellular LPS-receptor, in macrophages [40]. We show, for the first time to our knowledge, an interaction of bEV-localized LPS and macrophage-localized TLR4 with the fluorescence-based PLA. As both molecules must be in close proximity (≤ 40 nm) during the assay for colocalization signals to appear, we interpret these results as evidence of close spatial association between bEV-localized LPS and TLR4. Moreover, blocking TLR4 receptor recognition capabilities on macrophages with the antagonistic LPS-RS did not induce *ISG20* when macrophages were stimulated with bEVs from *Ec*. This finding functionally supports the role for TLR4 in the immunostimulatory properties of bEVs on macrophages. These results are consistent with and further support other publications that have consistently observed diminished or reduced bEV-stimulatory effects in TLR4 knockout cells or mice [17, 32, 41, 42]. This effect was also observed in previous work from our group, where we were able to inhibit influenza A and VSV replication in bEV-stimulated macrophages, which was restored to unstimulated cell levels in bEV-stimulated TRIF knockout cells [22].

We next wondered if macrophage activation by bEVs is effective enough to interfere with viral replication in lung epithelial cells. To this end, we exposed Calu-3 cells, a lung epithelial cell line susceptible to infection with SARS-CoV-2, to conditioned supernatants of bEV-stimulated macrophages. This resulted in the expression of multiple ISGs in the Calu-3 cells, with particularly strong induction of *Mx1* and *IFIT1*. Additionally, we found that ISG induction was blocked when cells were exposed to the JAK inhibitor Ruxolitinib, supporting an important role for IFNs in this propagation of an immune response. SARS-CoV-2 has been shown to be highly susceptible to interferon-mediated immune responses, significantly limiting its replication and spread [43]. Our findings in Calu-3 cells exposed to conditioned supernatants from bEV-stimulated macrophages further support this viral vulnerability. SARS-CoV-2 virus replication was reduced in Calu-3 cells exposed to the supernatants of bEV_*Kp*_- and bEV_*Ec*_-stimulated macrophages, which were the supernatants with the highest measured levels of cytokine secretion and cellular induction of ISGs. Although minimal vesicle carryover in macrophage-conditioned supernatants cannot be formally excluded, several lines of evidence argue against a direct contribution of epithelial bEV stimulation on the antiviral effect. We have previously shown that Calu-3 cells do not directly respond with induction of ISGs with the bEVs used here [22]. Moreover, in the present study epithelial ISG induction and antiviral effects were strictly dependent on interferon-JAK/STAT signaling, as demonstrated by Ruxolitinib-mediated inhibition. Together, these observations support a model in which macrophage-derived soluble mediators, rather than direct vesicle transfer, drive epithelial antiviral priming. While our data support the existence of a functional TLR4-IFN signaling axis linking bEV sensing in macrophages to epithelial antiviral responses, detailed resolution of downstream signaling nodes and individual IFN effector mechanisms remain to be investigated.

The choice of epithelial model represents an important consideration for interpreting these findings. Calu-3 cells were selected because they constitute one of the most widely used and best-validated human airway epithelial models for SARS-CoV-2 infection, as they endogenously express key viral entry factors and support robust, productive viral replication [44–46]. This makes them particularly well suited to assess IFN-dependent antiviral effects in an infection-relevant epithelial context. Importantly, prior work from our group demonstrated that direct exposure of multiple human airway epithelial cell lines, including A549, HCC827, Calu-3, and BEAS-2B cells, to the same panel of bEVs used here does not robustly induce IFN-stimulated genes [22]. These observations indicate that airway epithelial cells are not primary sensors of bEV-associated interferon-inducing signals and support the experimental focus on macrophage-mediated sensing and intercellular immune communication in the present study. Accordingly, epithelial antiviral responses observed here arise indirectly through macrophage-derived IFNs and should be interpreted within the context of the Calu-3 model and its suitability for interrogating IFN-dependent antiviral effects rather than direct bacterial sensing.

While secondary bacterial infections following primary viral infections are well recognized to worsen outcomes in viral pneumonia [7], data on secondary viral infections following bacterial infection remain scarce [47]. The experimental sequence used here does not represent a classical viral-bacterial co-infection model, such as influenza infection followed by secondary bacterial pneumonia, but was intentionally designed to address a distinct mechanistic question: how prior macrophage sensing of bacterial EVs shapes subsequent antiviral responsiveness in lung epithelial cells. This experimental system does not aim to recapitulate physiological infection chronology, but rather to dissect macrophage-driven intercellular signaling effects under controlled conditions. Primary bacterial infections directly preceding viral infection are comparatively rare in clinical settings and difficult to capture systematically, particularly in pulmonary disease. Our experimental approach provides a controlled framework to investigate how prior macrophage exposure to bacterial products including PAMP-containing bEVs, shapes subsequent interferon-dependent antiviral responsiveness in lung epithelial cells. These findings should therefore be interpreted within the limitations of an in vitro model.

Together, these findings support the broader concept that bacterial-derived immune activation, including via bEVs, may contribute to induction of interferon-associated antiviral states across interconnected immune and epithelial compartments.

This study specifically investigates the effect of bEV-mediated macrophage activation on a specific virus known to be strongly affected by interferons, it does not allow predicting the broader context of the immunostimulatory properties of bEVs during viral infections. However, as we have previously published results showing that bEV-activated macrophages have a similar inhibitory effect on viruses from two other families, namely influenza A virus and vesicular stomatitis virus [22], we suggest that a general mechanism of immune activation may be involved. This would also be consistent with the general knowledge of interferons and their complex interactions, as they have been found to induce more than 300 ISGs, which in turn provide a variety of functions [48–50]. Co-infection experiments with SARS-CoV-2 and *Mycobacterium tuberculosis* in mice revealed a limited viral replication and improved survival rates in the presence of bacteria, possibly mediated by bEVs [51, 52]. Although bEVs were not the focus of these publications, macrophages infected with *Mycobacterium tuberculosis* were reported to release extracellular vesicles resembling bEVs, and cell wall components of these bacteria are known to induce diverse immune responses even in non-infected bystander cells [53–57].

Comparative studies of bEVs from different origins, as performed in this study, could help to distinguish between species-specific immunostimulatory effects and general properties of bEVs. Beyond the respiratory system, immunomodulatory effects of bEVs have been described in multiple biological contexts, including modulation of host immune responses in the gut, systemic circulation, and infection models involving diverse host–pathogen interactions. These observations indicate that bEV-mediated immune activation represents a broadly conserved mechanism by which bacteria influence host immunity across tissues [58–60]. Vesicles might prove useful in the development of new therapeutics or vaccines, as they can present antigens in a native membrane-anchored configuration and co-display multiple immune system agonists on the same particle in close proximity, potentially resulting in more favorable immune activation patterns [61]. Notably, a bEV-based vaccination strategy using *Neisseria meningitidis*-derived bEVs, linked to a recombinant SARS-CoV-2 spike protein protected Syrian hamsters against viral infection, particularly with intranasal administration [62]. However, these approaches carry the inherent risk of excessive activation and damage to immune cells and require a thorough understanding of the immune responses involved [63].

Despite their immunostimulatory potential, native bEVs also present inherent challenges for translational application. The presence of LPS and the heterogeneity of vesicle cargo may lead to excessive or uncontrolled inflammatory responses and complicate standardization across preparations. Consequently, direct therapeutic or vaccine use of unmodified bEVs would require careful consideration of safety, dosing, and reproducible manufacturing, as variability in vesicle composition may complicate standardization across preparation. Future strategies aiming to harness bEV-derived immunostimulatory principles will likely depend on engineered or detoxified vesicle platforms, or on synthetic systems that recapitulate defined immunogenic features while minimizing adverse inflammatory effects, rather than direct application of native bEV preparations.

Several limitations of the present study should be acknowledged. First, all experiments were performed in vitro using primary human macrophages and the Calu-3 epithelial cell line, which cannot fully recapitulate the cellular complexity and intercellular communication of the infected lung. Second, while our data support a TLR4-dependent macrophage activation pathway and subsequent interferon-associated epithelial responses, the individual soluble mediators responsible for epithelial priming were not dissected in detail. Third, the study was designed to compare bEVs from bacterial species with distinct immunostimulatory properties rather than to model clinically occurring co-infection scenarios. Finally, although reduced SARS-CoV-2 replication was observed in response to conditioned supernatants from bEV-stimulated macrophages, the mechanistic contribution of individual interferons and downstream effector molecules remains to be determined.

As macrophages are often one of the first cells of the immune system to get in contact with pathogens and initiate immune responses, they remain one of the most important cell types for studying the innate immune response and deepening our understanding of pathogen-host interactions. With this study, we provide a deeper mechanistic understanding of how bEVs shape innate immune communication between macrophages and epithelial cells. Future studies should investigate whether similar mechanisms operate in more complex experimental systems, including primary airway models and in vivo infection settings, and identify the specific vesicle-associated molecules responsible for these effects.

A better understanding of these pathways may support the development of bEV-inspired immunomodulatory approaches, vaccine platforms, or host-directed therapeutic strategies aimed at enhancing antiviral defense mechanisms.

## Supplementary Information

Supplementary Material 1.

## Data Availability

The datasets generated and analyzed during the current study are included in the manuscript and its supplementary information files. Additional data are available from the corresponding author upon reasonable request.

## Abbreviations

BDMs: Blood-derived macrophages
bEVs: Bacterial extracellular vesicles
BYE: Buffered yeast extract broth
CD14: Cluster of differentiation 14
CXCL8: Chemokine (C-X-C motif) ligand 8
DAPI: 4´,6-Diamidin-2-phenylindol
Ec: Escherichia coli
hGM-CSF: Human granulocyte macrophage colony-stimulating factor
IFIT1: Interferon-induced protein with tetratricopeptide repeats 1
IFN: Interferon
IFN-β: Interferon-β
IL-1β: Interleukin-1β
IL-8: Interleukin 8
ISG20: Interferon-stimulated gene 20 kDa
Kp: Klebsiella pneumoniae
Lp: Legionella pneumophila
LPS: Lipopolysaccharides
LPS-RS: Lipopolysaccharides from Rhodobacter sphaeroides
MOV: Multiplicity of vesicles
Mx1: Interferon-induced GTP-binding protein Mx1
nFCM: Nano Flowcytometry
OASL: Oligoadenylate synthase-like protein
PAMPs: Pathogen associated molecular patterns
PMA: Phorbol 12-myristate 13-acetate
RPS18: Ribosomal protein S18
Sal: Salmonella enterica serovar Typhimurium
SARS-CoV-2: Severe Acute Respiratory Syndrome-Coronavirus-2
SEC: Size-exclusion chromatography
Sp: Streptococcus pneumoniae
TLR4: Toll-like receptor 4
