# Supplementary material for: Bacterial extracellular vesicles modulate epithelial antiviral responses via macrophage-mediated immunomodulation
Source: Cell Commun Signal. 2026 Jun 24;24:371. doi: 10.1186/s12964-026-03003-x (PMC13292577; doi:10.1186/s12964-026-03003-x)
Supplement: Supplementary file 1 — Supplementary Material 1. [file 12964_2026_3003_MOESM1_ESM.docx]

**Supplement**

**Bacterial extracellular vesicles modulate epithelial antiviral responses via macrophage-mediated immunomodulation**

**Supplementary Figure 1**


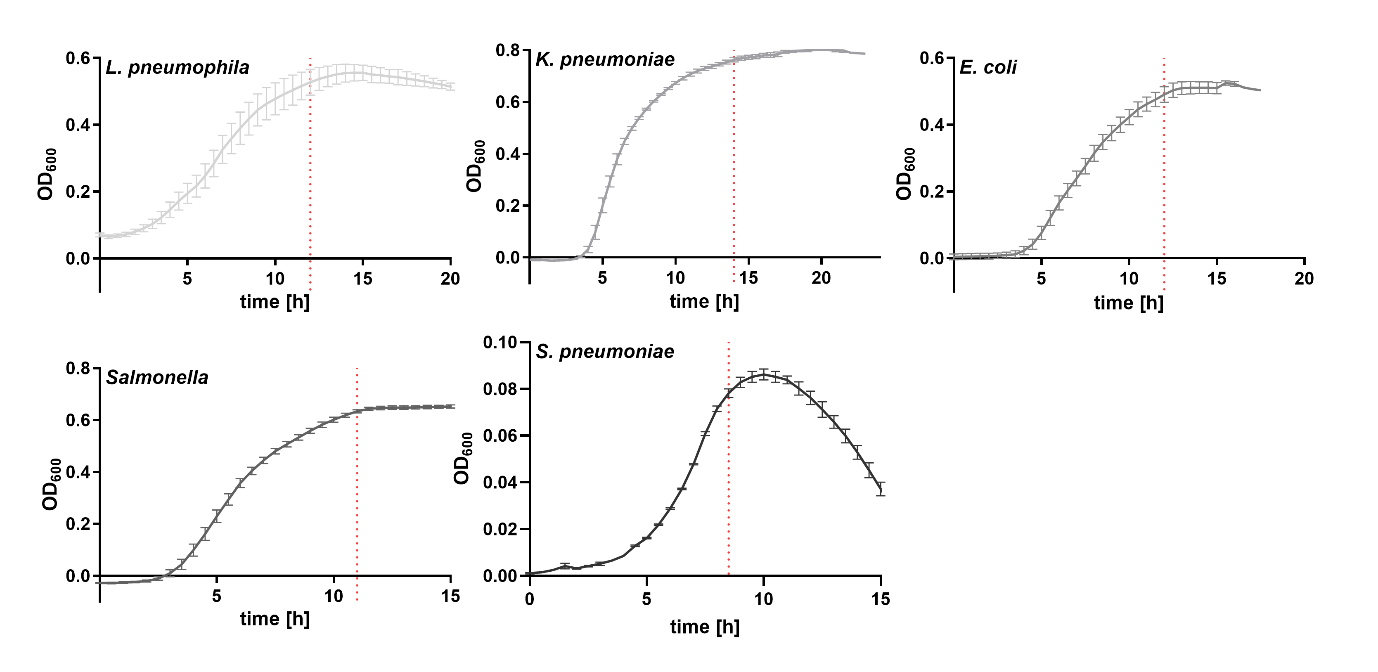


**Supplementary Figure 1: Growth curves of bacterial strains used for bEV isolation.** Bacterial growth curves of *Legionella pneumophila* (*Lp*), *Klebsiella* *pneumoniae* (*Kp*), *Escherichia* *coli* (*Ec*), *Salmonella* *enterica* serovar Typhimurium (*Sal*), and *Streptococcus* *pneumoniae* (*Sp*) under species-specific culture conditions used for bEV production. Optical density (OD) at 600 nm was measured over time to monitor bacterial growth. Dashed lines indicate the time points at which cultures were harvested for bEV isolation. Sampling time points were adjusted for each bacterial species to ensure comparable growth phases at the time of vesicle collection. Data represent mean ± SEM of three biological replicate.

**Supplementary Figure 2**


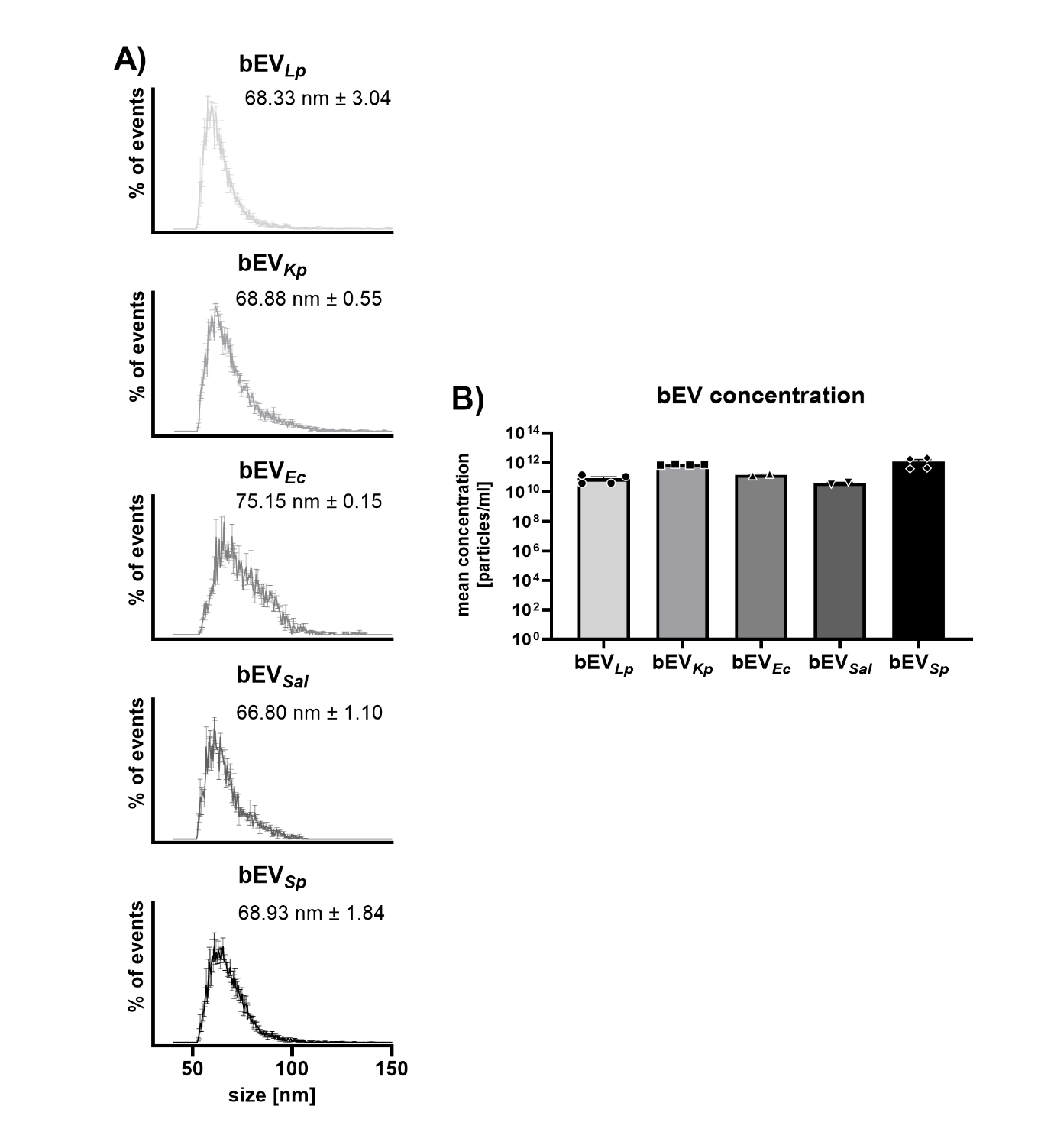


**Supplementary Figure 2: NanoFCM characterization of bacterial extracellular vesicle preparations.** Bacterial extracellular vesicles were prepared from *Legionella pneumophila* (bEV*_Lp_*), *Klebsiella pneumoniae* (bEV*_Kp_*), *Escherichia coli* (bEV*_Ec_*), *Salmonella* Typhimurium (bEV*_Sal_*) and *Streptococcus pneumoniae* (bEV*_Sp_*). (A) NanoFCM size distribution profiles of bEV preparations derived from 2-4 independent biological replicates. Curves represent mean values and error bars indicate ±SEM. Mean vesicle size ±SEM calculated from the corresponding measurements is indicated within each graph. (B) Mean vesicle concentration of bEV preparations as determined by nFCM. Bars represent the mean value ±SEM of two to four biological replicates. Symbols represent replicates.

**Supplementary Figure 3**

**
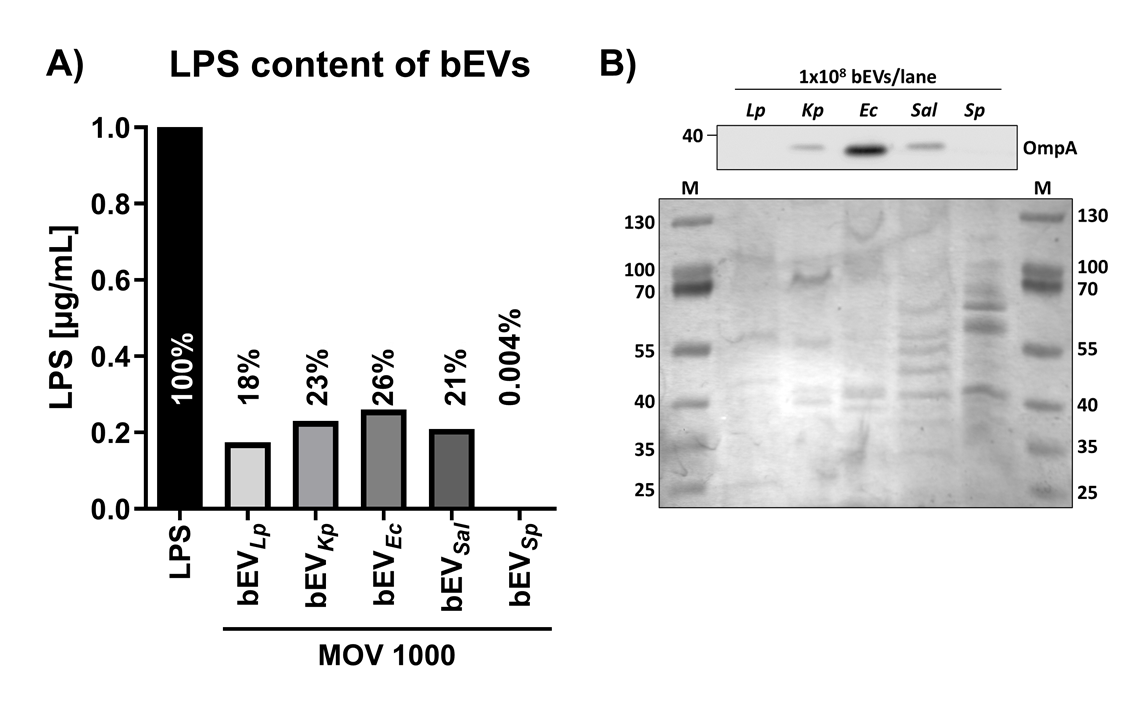
**

**Supplementary Figure 3**: **Characterization of bEV preparations.** (A) Limulus Amebocyte Lysate (LAL) assay quantifying endotoxin content in bEV preparations derived from *Legionella pneumophila* (bEV*_Lp_*), *Klebsiella pneumoniae* (bEV*_Kp_*), *Escherichia coli* (bEV*_Ec_*), *Salmonella* Typhimurium (bEV*_Sal_*), and *Streptococcus pneumoniae* (bEV*_Sp_*). Endotoxin levels are shown relative to a commercially available *E.coli* LPS standard. Values represent the percentage of LPS detected in bEV preparations relative to the LPS control. Bars indicate mean values. (B) Western blot analysis for outer membrane protein A (OmpA) in bEV preparations (upper panel). Equal numbers of vesicles ($1\cdot{10}^{8}$ bEVs) were loaded per lane. A representative blot is shown. Lower panel shows a Coomassie-stained gel. M = molecular weight marker.

**Supplementary Figure 4**


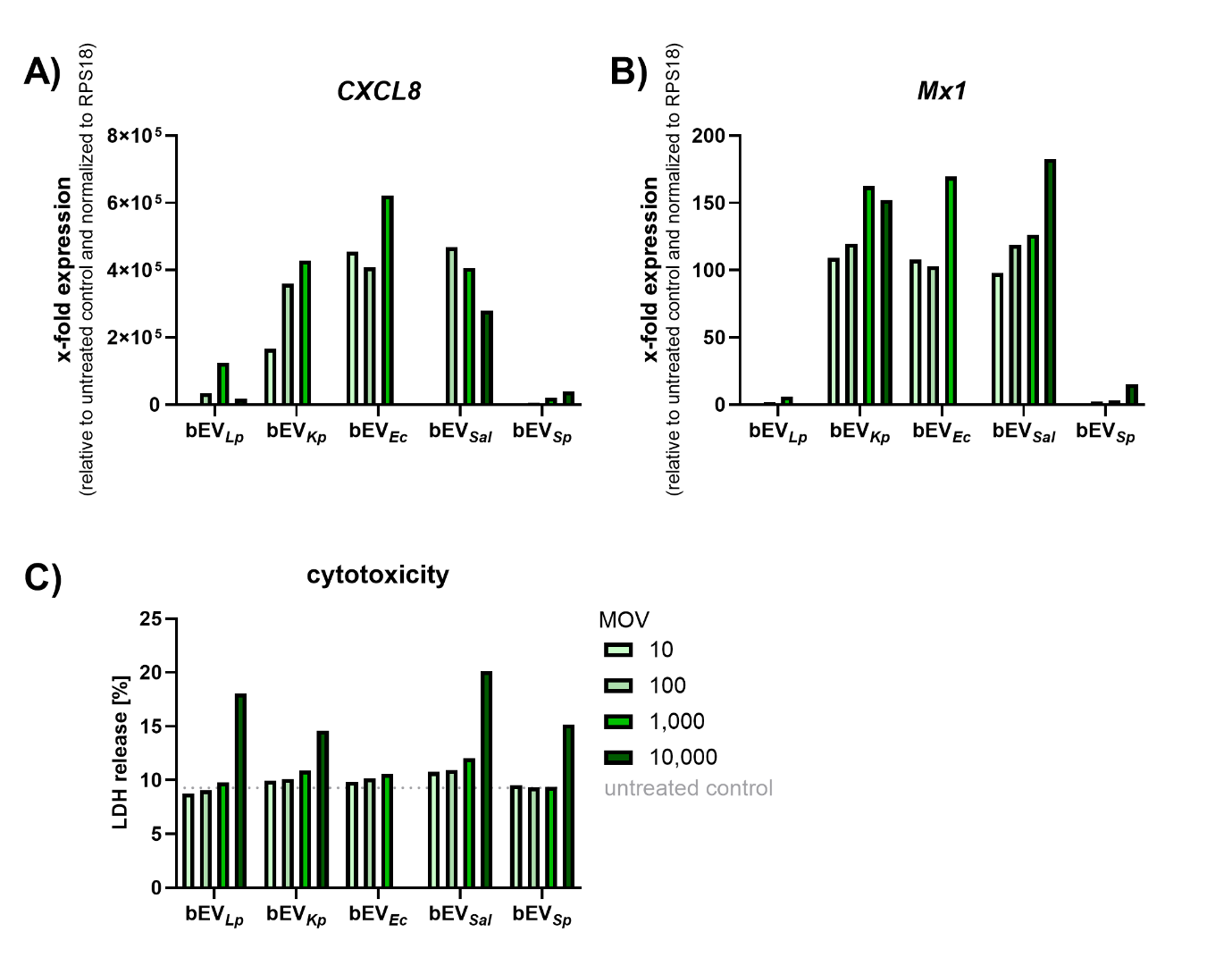


**Supplementary Figure 4: Dose-response analysis of bEV stimulation in primary human macrophages.** Primary human blood-derived macrophages (BDMs) were stimulated with increasing multiplicities of vesicles (MOV 10, 100, 1,000, and 10,000) for 20 h for all bEV preparations.) Relative gene expression of CXCL8 (A) and Mx1 (B) assessed by qPCR. (C) Cytotoxicity determined by lactate dehydrogenase (LDH) release in cell culture supernatants. Dashed line indicates untreated control cells. Data represent mean values from two independent donors.

**Supplementary Figure 5**


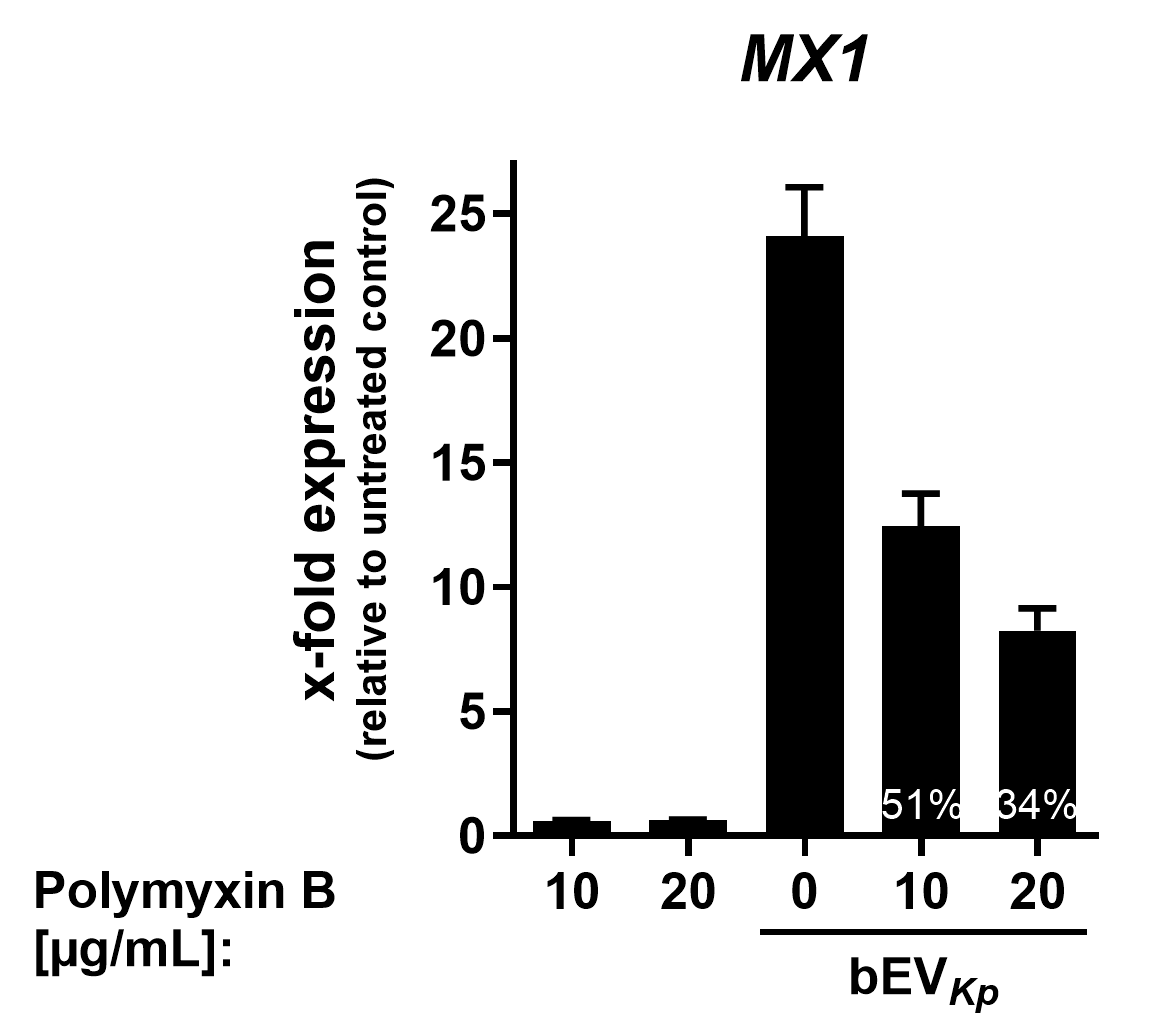


### Supplementary Figure 5: Polymyxin B-mediated neutralization of bEV-induced macrophage activation. THP-1-derived macrophages were stimulated with *Klebsiella pneumoniae*-derived bEVs (bEV*_Kp_*) in the presence or absence of polymyxin B (PB; 10 and 20 µg/mL) for 20 h. Relative Mx1 expression was assessed by qPCR. PB treatment reduced bEV-induced Mx1 expression in a concentration-dependent manner, consistent with partial neutralization of LPS activity. Data represent mean values from three independent experiments.

**Supplementary Figure 6**

*_
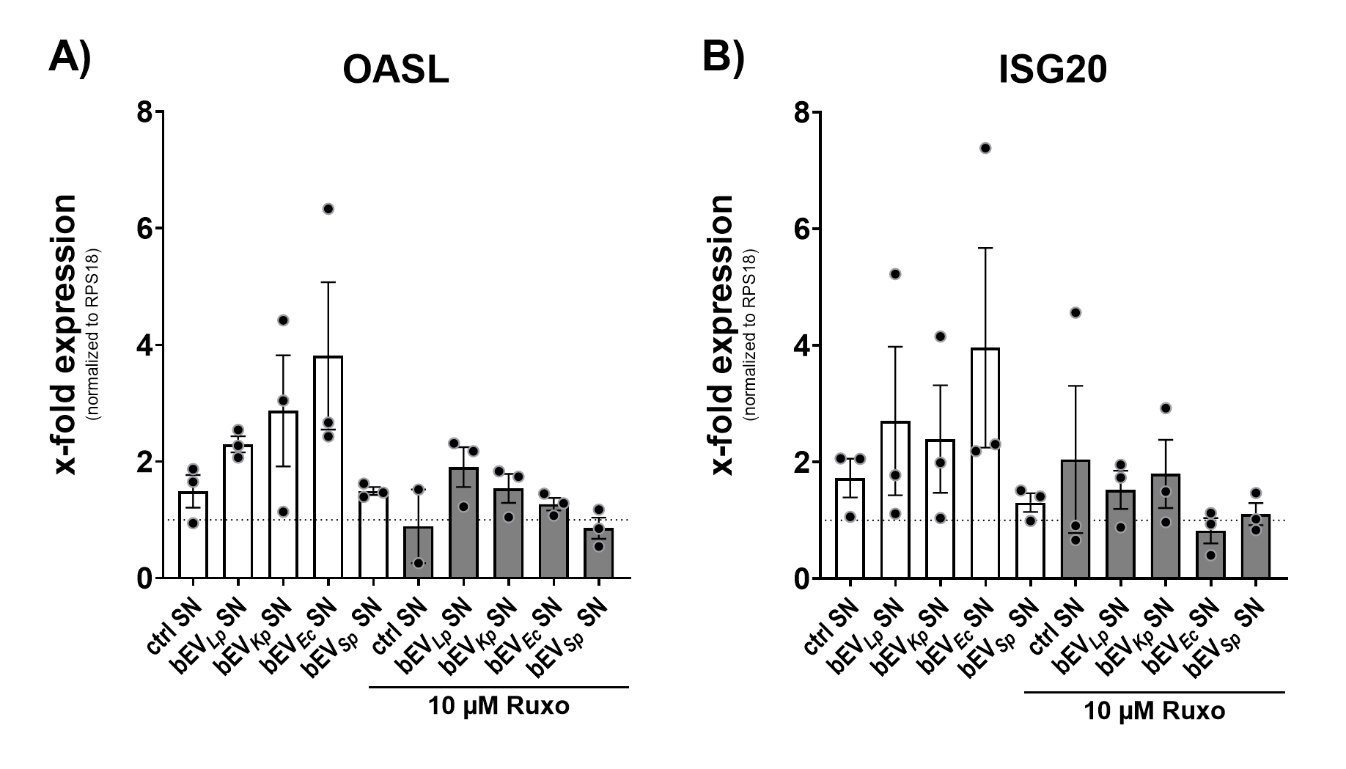
_*

**Supplementary Figure 6:** **ISG expression in Calu-3 cells following stimulation with conditioned supernatants from bEV-treated BDMs.** (A+B) Calu-3 cells were stimulated for 20 h with conditioned supernatants obtained from blood-derived macrophages (BDMs) stimulated with bacterial extracellular vesicles from *Legionella pneumophila* (bEV*_Lp_* SN), *Klebsiella pneumoniae* (bEV*_Kp_* SN), *Escherichia coli* (bEV*_Ec_* SN), or *Streptococcus pneumoniae* (bEV SN), or from unstimulated control BDMs (Ctr SN), as indicated on the x-axis. Where indicated, Calu-3 cells were additionally treated with 10 µM Ruxolitinib (Ruxo). Relative expression of OASL (A) and ISG20 (B) was determined by qPCR and normalized to RPS18. Data are shown as x-fold change relative to untreated Calu-3 cells, which were used as the normalization reference and are indicated by the dashed line at y = 1. Bars represent mean ± SEM from three independent experiments. Symbols indicate biological replicates derived from independent BDM donors.

Statistics: One-way ANOVA followed by Tukey’s multiple comparisons test. Statistical comparisons were performed between corresponding conditioned supernatant treatments in the absence or presence of 10 µM Ruxolitinib (Ruxo). No statistically significant differences were observed.

**Supplementary Figure 7**


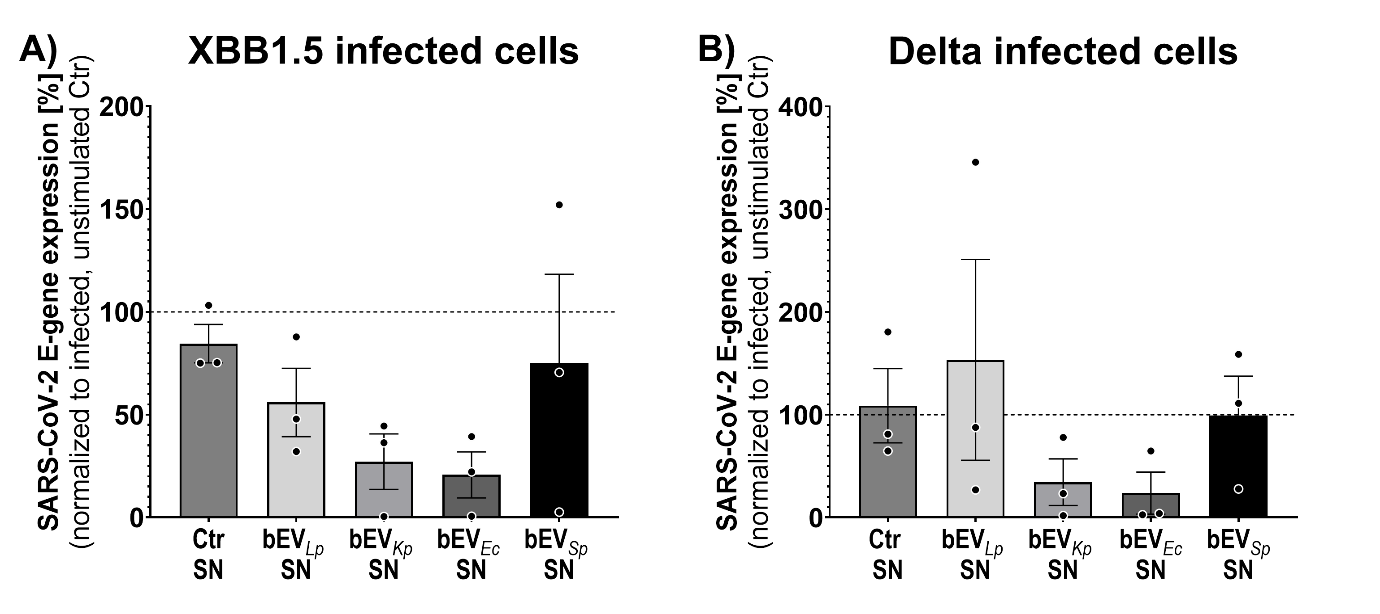


**Supplementary Figure 7: Interference of SARS-CoV-2 replication in infected Calu-3 cells, after pre-stimulation with supernatants from bEV-stimulated BDMs.** (A+B) SARS-CoV-2 E gene expression was determined by qPCR after infection of Calu-3 cells with SARS-CoV-2 XBB1.5 (A) or Delta (B) variants for 24 h at an MOI of 0.01. Prior to infection, cells were stimulated for 20 h with supernatant from BDMs stimulated for 20 h with the indicated bEVs (*Legionella pneumophila* (bEV*_Lp_* SN), *Klebsiella pneumoniae* (bEV*_Kp_* SN), *Escherichia coli* (bEV*_Ec_* SN) and *Streptococcus pneumoniae* (bEV*_Sp_* SN)) or left untreated as control. Bars represent mean values ±SEM from three independent experiments, normalized to RPS18 and relative to untreated but infected Calu-3 control cells. Symbols represent replicates.
